# Infants' perception of lightness changes related to cast shadows

**DOI:** 10.1371/journal.pone.0173591

**Published:** 2017-03-15

**Authors:** Kazuki Sato, So Kanazawa, Masami K. Yamaguchi

**Affiliations:** 1 Department of Psychology, Chuo University, Hachioji, Tokyo, Japan; 2 Japan Society for the Promotion of Science, Tokyo, Japan; 3 Department of Psychology, Japan Women’s University, Kawasaki, Kanagawa, Japan; Durham University, UNITED KINGDOM

## Abstract

When humans perceive the lightness of an object’s surface in shadows there is an implicit assumption that cast shadows dim the surface. In two experiments, we investigated whether 5- to 8-month-old infants make this assumption about shadows. According to this shadow assumption, the apparent change in lightness produced by shadows on an object’s surface are attributed to blocked light sources. If infants can use the shadow assumption to perceive the object’s lightness in shadows, they will also be able to detect unnatural lightness changes in shadows. We compared the infants’ looking times to the unnatural and the natural lightness changes in the shadow when an object (duck) goes through the cast shadow. In Experiment 1, we examined whether infants could detect the unnatural lightness changes of the object’s surface in shadows. We created computer-graphic movies of unnatural and natural lightness changes to the duck’s surface. Our results showed that 7- to 8-month-olds but not 5- to 6-month-olds significantly preferred the movie with the unnatural changes in lightness, indicating that only the older infants could detect these changes. In Experiment 2, we confirmed that the infants’ preference was based on the detection of unnatural lightness changes according to the shadow assumption. The natural and the unnatural lightness changes of Experiment 1 were presented without cast shadows. Under these conditions, neither younger nor older infants showed a significant preference. Taken together, the experiments showed that 7- to 8-month-old infants could detect the unnaturalness of a surface’s lightness changes produced by shadows. In conclusion, our findings suggest that 7- to 8-month-old infants can perceive an object’s lightness in shadows by using an assumption that cast shadows dim the surface of an object.

## Introduction

Shadows are fundamental components of natural visual scenes. When the illumination source of a seen object is obstructed, a dark region is projected onto the object’s surface; this region is called a cast shadow. Humans can rapidly judge whether a dark area on an object’s surface is a cast shadow or not (cf. [1]). When humans observe an object passing through cast shadows, they can perceive that the changes in lightness of the object’s surface are caused by blocked light sources and not by changes in reflectance properties of the object’s surface; i.e., the objects are perceived to have lightness constancy [2]. The assumption is that shadows cast on an object will dim the object’s surface. In the current study, we investigated whether infants employ this assumption.

Previous studies [3–5] examined whether 4-month-old infants exhibit the ability of lightness constancy. Chien et al. [5] tested infants’ responses to three factors: (1) an object’s luminance, (2) an object’s reflectance and (3) the local luminance ratio of adjacent regions. To behave under a lightness-constant manner, humans should detect that the luminance ratio of adjacent regions is constant despite various illuminations [6]. The results showed that infants had sensitivity to local luminance ratios as cues to stable perception of lightness of a surface. The study confirmed that 4-month-old infants could use local luminance ratios as an important cue to the presence of lightness constancy. They also revealed that 4-month-old infants could show deviations from the ratio rule, just as adults do [4]. However, the mechanisms and cues for lightness constancy in adults are still under debate [7]. Many illusions, e.g., White’s illusion [8, 9], show that local contrast is not the only cue to lightness constancy [10–12]. An infant study found that the chromatic version of White’s illusion [8], called the Munker illusion, could be perceived by 4- to 8-month-old infants. Perception of White’s illusion in adults and infants suggested that both can use the same method for perceiving lightness. However, it is an open question whether sensitivity to lightness constancy differs between adults and infants.

In the current study, we capitalized on infants’ preference for “unnaturalness” (impossibility) to test whether they could detect unnatural changes in lightness produced by cast shadows. Previous studies have established a precedent for using this unnaturalness preference to investigate infants’ perception [13–15]. For example, Shuwairi [15] tested whether infants would prefer a three-dimensionally impossible cube to a three-dimensionally possible version. These cubes were depicted by line junctions, and the impossible cube had a structurally incoherent shape. Results showed that 4-month-old infants preferred the impossible cubes, which suggested that these infants can perceive 3D structures from line junctions. Csibra [13] also examined whether infants preferred deletion and accretion patterns of a moving duck that were consistent or inconsistent with the subjective contours’ depth. The 8-month-old infants preferred the inconsistent deletion and accretion patterns, prompting the conclusion that these infants can perceive subjective contour as an object acting as an occluder. Infant studies on cast shadow have also used the preference for unnaturalness [16]. Van de Walle and her colleague [16] demonstrated that 5- and 8-month-old infants could detect unnaturalness from unsynchronized motion of a shadow. In their experiments, adults rated unnaturalness of static or moving scenes that comprised three elements: a ball, its cast shadow, and a floor. The researchers used these scenes to conduct three infant experiments. In each experiment, the infants first learned a natural scene in habituation trials, then were exposed to test trials. In the first experiment, after learning a static natural scene, the infants preferred the unnatural version of an analogous moving scene, in which a ball was not synchronized with its cast shadow. Experiments 2 and 3 both used a synchronization condition and a naturalness condition. In the synchronization condition, they presented a natural synchronized moving scene or an unnatural unsynchronized moving scene. In the naturalness condition, they presented a natural unsynchronized moving scene versus an unnatural unsynchronized moving scene. In Experiment 2, infants learned a natural static scene as in Experiment 1. In Experiment 3, they learned a natural moving scene. Experiment 2 showed that infants preferred the natural moving scene in both synchronization and naturalness conditions. Experiment 3 showed that infants only preferred the unnatural moving scene in the synchronization condition. Therefore, Experiments 2 and 3 suggested that infants can detect the unnaturalness of cast shadows by using synchronicity of motion of objects with their shadows. In this study, we used a preference for unnaturalness to examine infants’ perception of lightness change related to cast shadows.

The current study investigated whether 5- to 8- month-old infants could detect unnatural changes in a surface’s lightness produced by cast shadows; that is, we tested whether infants used the assumption employed by adults, that the lightness of an object’s surface is reduced when the object passes through shadows. We created a Computer Graphic (CG) movie that depicted a duck passing into and out of shadows, where the lightness of the duck’s surface was reduced whenever the duck was moving through the cast shadows. To create the unnatural movie, we reversed the lightness of the duck’s surface inside and outside of the shadows. We collected the data from 5- to 8-month-old infants as was done in Csibra’s study [13], using an unnatural preference. We hypothesized that if infants were using the assumption that an object’s surface becomes darker when shadowed, they would detect the unnatural change in lightness, and this detection would be demonstrated by a preference for the unnatural movie.

## Experiment 1

### Materials and methods

#### Participants

Participants were 16 5- to 6-month-old infants (6 males, mean age 163.94 days, range 146 to 188 days), and 16 7- to 8-month-old infants (8 males, mean age 224.69 days, range 196 to 251 days). All of the infants were full-term at birth. According to Csibra's unnatural preference experiment [13], this age is suitable for this experimental procedure.

An additional 13 infants were tested in this experiment, but their data were excluded from analysis owing to fussiness (n = 2), or longer looking times in the last three familiarization trials than in the first three (n = 11).

This study was approved by the Ethical Committee of Chuo University (2014–1, 2015–13). Written informed consent was obtained from the parents of the infant participants.

#### Apparatus

Throughout the experiments, all stimuli were displayed on a 21-inch color CRT monitor. The infant and CRT monitor were located inside an enclosure made of plastic poles covered with black cloth. Each infant sat on the lap of his/her parent in front of a monitor. The distance between the infants and the monitor was approximately 40 cm. One speaker was positioned on each side of the CRT monitor. There was a pinhole CCD camera just below the monitor screen. Throughout the experiment, the infant’s behavior was videotaped through this camera. The experimenter could observe the infant’s behavior via a monitor connected to the pinhole camera.

The experiment was programmed in Python 2.5, with the use of Vision Egg 2.3. Stimuli were constructed off-line before the experiment using 3D rendering software (New Tec LightWave 11.0) and the computer graphic software (Adobe Photoshop CS 6).

#### Stimuli

**Fig 1:**
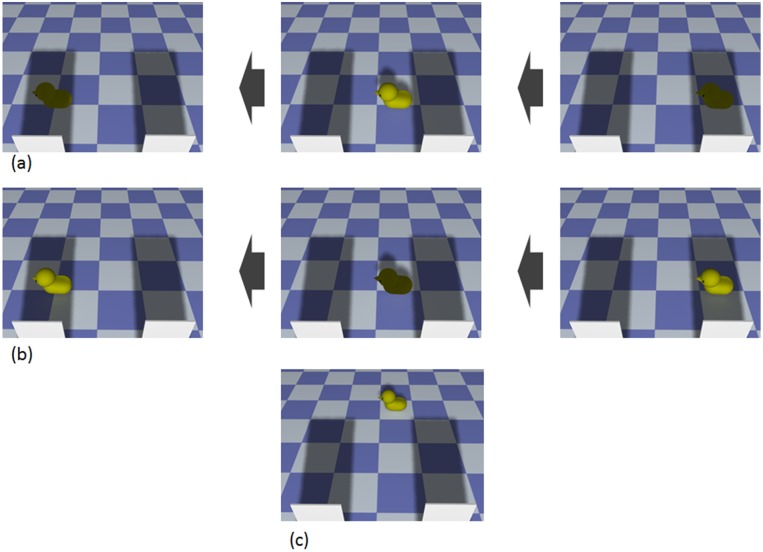
Stimuli of Experiment 1. (a) Example of the natural condition: the lightness of the duck’s surface was darker in cast shadows. (b) Example of the unnatural condition: the lightness of the duck’s surface becomes lighter in cast shadows and darker outside of cast shadows. (c) Example of the familiarization phase: the duck moved across the floor without entering the cast shadows. The procedure for generating movies was as follows: We created 3DCG of a duck going through the two panels’ cast shadows. The no-lightness-change movie was rendered from the 3DCG, excluding the shadow that was cast on the duck’s surface, so that the lightness of the duck’s surface was constant. We placed shadows on the no-lightness-change duck to change the duck’s lightness naturally and unnaturally. We used LightWave 11.5 to create 3DCGs and to render 3DCGs as bitmap files. We edited image files with Photoshop CS6.

We created three types of CG movies; two were used for test trials (natural condition and unnatural condition) and the other was used for familiarization trials (Fig 1). First, we created a display where two panels stood upright at the front of a checkered floor. Each panel cast a cast shadow on the floor. In all movies, a yellow duck passes through the panels’ cast shadows from right to left, always at the same velocity (7.42 deg/s). Width and height of all movies subtended 34.70 degrees and 25.36 degrees of visual angle, respectively.

In the natural condition, the lightness of the duck’s surface changed as it would normally: when the duck passed through the cast shadow, the lightness of its surface was reduced. In the unnatural condition, the lightness of the duck’s surface increased when the duck was in the cast shadows, and decreased when the duck was outside of the cast shadows. In both conditions, the mean luminance values of the lighter surface (mean luminance = 21.96 cd/m^2^) and of the darker surface (mean luminance = 2.89 cd/m^2^) were held constant.

For the familiarization trial, the duck’s trajectory was shifted upward 150 pixels from test trials, so that it passed across the floor from right to left without entering the panels’ cast shadows. In these trials, the lightness of the duck’s surface was constant (mean luminance = 21.96 cd/m^2^). The mean of Michelson contrast was 92.50% (*SD* = 14.05) for natural condition and 92.38% (*SD* = 14.07) for unnatural condition.

#### Procedure

At the outset of each trial, a cartoon with a short sound was presented at the center of the monitor to get the infant’s attention. The experimenter initiated the trial as soon as the infant looked at the cartoon. In each trial, one movie played in a loop at the center of the monitor. When the trial terminated, the movie stoped playing.

First, experimenters presented the familiarization movie for six trials. All of these familiarization trials were terminated when the infant looked away from the monitor for at least 3 s, or when the trial reached a 15-s maximum. The familiarization trials were followed immediately by the two test trials (the natural and the unnatural condition). The order of the two test trials was counterbalanced across infants. Each test trial was presented for 30 s. During all trials, infants’ looking behavior was recorded digitally.

#### Data coding

One observer, who had no access to the stimuli the infants were viewing, measured each infant’s looking time to the stimuli from the recorded video. Observers were made to judge whether the infant looked at the stimuli, or did not look at the stimuli from observation of recorded videos. Observers depressed a key whenever an infant was looking at the stimuli, and they released the key whenever an infant looked away from the stimuli. Our program recorded the time that the key was depressed in milliseconds. We summed and defined it as the infants’ looking time. To calculate interobserver agreement, a second observer’s measurement of the infants’ looking time was obtained for 31.25% of the total data. An interobserver agreement was *r* = .971.

### Results

#### Familiarization trials

For the familiarization trials, individual looking times were averaged across the first three and the last three trials (Table 1). We performed a 2×2 ANOVA for the looking times, with (i) trial (the first three or the last three) as a within-participant factor and (ii) age group (5- to 6- month-olds or 7- to 8-month olds) as a between-participants factor. The ANOVA revealed a significant main effect of trial: *F*(1,30) = 59.46, *p* < 0.001, η_p_^2^ = 0.66, which reflects a decrease in looking times over the trials. No other effects were reliable: *F*(1,30) = 0.78, *p* = 0.38, η_p_^2^ = 0.03 (main effect of age group), *F*(1,30) = 0.03, *p* = 0.87, η_p_^2^ < 0.001 (interaction of trial and age group). These results confirmed that infants became more familiar with the stimuli over the six trials, and that this familiarity did not differ by age group

**Table 1:** Mean total looking time (in seconds) in the familiarization trials and test trials. Standard deviations are given in parentheses.

	Familiarization trials				Test trials (max = 30s)	
	First three trials (max = 15s)		Last three trials (max = 15s)			
Age group	Mean (s)	SD	Mean (s)	SD	Mean (s)	SD
5-to-6-month olds (n = 16)	12.52	1.55	10.26	2.27	21.13	5.52
7-to-8-month olds (n = 16)	13.12	1.18	10.76	2.51	23.45	3.88

#### Test trials

The mean total looking times of each age group were 21.13 s for 5- to 6- month-olds, and 23.45 s for 7- to 8-month-olds (Table 1). In the natural condition, the mean total looking times were 21.29 s for the younger group, and 21.42 s for the older group. In the unnatural condition, the mean total looking times were 20.98 s for the younger infants, and 25.47 s for the older ones.

**Fig 2:**
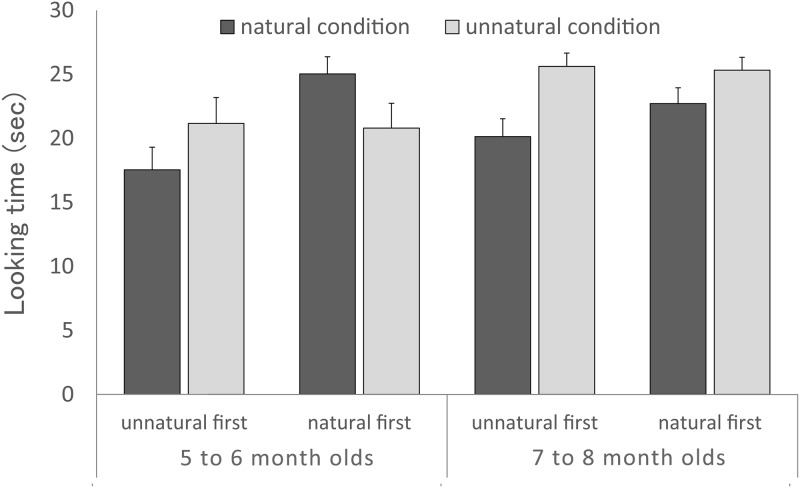
Results of Experiment 1. Mean looking times of infants for each condition as a function of age group and presentation order. Error bars indicate standard errors.

The looking times during the test trials are depicted in Fig 2. To examine whether infants looked longer at the unnatural condition than the natural condition, a 2×2×2 ANOVA was performed on preference scores, with (i) condition (natural or unnatural) as a within-participants factor and (ii) age group (5 to 6 months or 7 to 8 months) and (iii) order of test trial (natural condition first or unnatural condition first) as between-participants factors. The ANOVA revealed a significant main effect of condition: *F*(1, 28) = 13.31, *p* = 0.001, η_p_^2^ = 0.32. No other main effects were reliable (*p* > 0.05): *F*(1,28) = 2.64, *p* = 0.16, η_p_^2^ = 0.09 (main effect of condition), *F*(1,28) = 2.72, *p* = 0.11, η_p_^2^ = 0.09 (main effect of order of test trial). The other statistically significant outcomes were the interaction of condition and age group: *F*(1, 28) = 18.02, *p* = 0.001, η_p_^2^ = 0.39; the interaction of condition and order of test trial: *F*(1, 28) = 27.39, *p* < 0.001, η_p_^2^ = 0.50; and the three-way interaction: *F*(1, 28) = 5.91, *p* = 0.02, η_p_^2^ = 0.50 (all other *p* > 0.05).

To explore what drove these interactions, we performed post-hoc analysis. The simple effects analyses showed that mean looking times of the 7- to 8-month-olds were significantly different in the natural (21.42 s) and the unnatural (25.47 s) conditions: *F*(1, 14) = 50.67, *p* < .0001, η_p_^2^ = 0.78. The looking times for the unnatural condition were also significantly different between the age groups: *F*(1, 14) = 8.12, *p* = 0.008, η_p_^2^ = 0.22. These results indicate that 7- to 8-month-olds preferred the unnatural condition.

The results of the simple effects analyses also revealed the significant effect of trial order, as follows: The mean looking times for the natural condition were significantly different with respect to order of test trial (natural-first = 23.87 s; unnatural-first = 18.84 s); *F*(1, 14) = 12.07, *p* = 0.002, η_p_^2^ = 0.30. These results show that infants looked longer at the natural condition when it was presented in the first trial. The mean looking times for the unnatural-first group were significantly different between the natural (18.84 s) and unnatural (23.39 s) conditions: *F*(1, 14) = 36.33, *p <* 0.001, η_p_^2^ = 0.72. Infants looked longer at the unnatural condition when it was presented in first trial. Taken together, the findings show that the order of test trial affected the infants' looking time. No other effects were reliable (*p* > 0.05).

In sum, these results reveal that infants generally looked longer at whichever test condition was presented first; however, only 7- to 8- month-old infants significantly preferred the unnatural condition regardless of presentation order. These results suggest that 7- to 8-month-old infants were able to detect the unnatural lightness changes in the duck’s surface in the shadows. Our stimuli for familiarization and test trials were different in some of the following respects: the size of the duck, the change in the duck’s lightness, the trajectory of the duck and the length of the movie. These differences should increase the preference for the trails; however, the same effect has been documented by Csibra [13], so this effect did not impair our results.

## Experiment 2

In Experiment 1, only 7- and 8-month-old infants showed a preference for lightening of the duck’s surface as it passed through the cast shadows. This result indicated that infants at this age could detect the unnatural lightness changes. However, there is a possibility that the infants’ preference in Experiment 1 was not based on shadow perception but on lightness changes without environmental context. We tested this possibility in Experiment 2 by removing the cast shadows of the panels from the displays in Experiment 1. In both the natural and unnatural conditions of Experiment 2, the luminance values of the duck’s surface were the same as in the original natural and unnatural conditions of Experiment 1.

### Materials and methods

#### Participants

Participants were 16 5- to 6-month-old infants (7 males, mean age 172.38 days, range 148 to 193 days), and 16 7- to 8-month-old infants (8 males, mean age 226.44 days, range 195 to 252 days). All infants were full-term at birth. Twenty-nine infants participated only in Experiment 2, and the other three infants participated in both Experiments 1 and 2.

An additional 7 infants were tested in this experiment, but their data were excluded from analysis due to fussiness (n = 1), or longer looking times in the last three familiarization trials than in the first three (n = 6).

#### Stimuli

**Fig 3:**
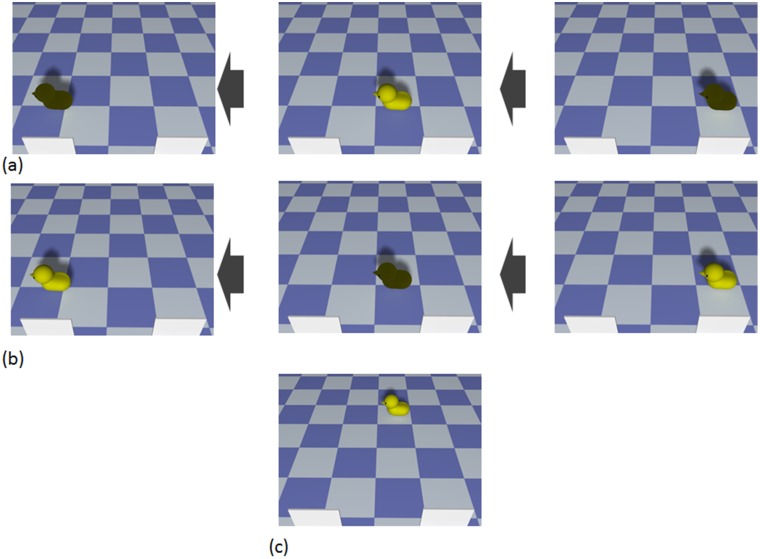
Stimuli of Experiment 2. (a, b) Examples of the natural (a) and unnatural (b) conditions without context: the lightness changes to the duck were the same as in Experiment 1, but the panels’ cast shadows were removed. (c) Example of the familiarization trials: the duck moves across the floor as in Experiment 1. Based on the Experiment 1’s 3DCG of a duck going through the two panels’ shadows, we created (a) and (b) movies. The procedure for generating movies was as follows: We rendered the movie excluding the shadows of the panels, so that the panels stood on the floor without cast shadows. To change the lightness of the duck’s surface as had been done in the natural and the unnatural conditions of Experiment 1, we placed shadows on the surface of the duck. We used LightWave 11.5 to create 3DCGs and to render 3DCGs as bitmap files. And we edited image files by using Photoshop CS6.

The stimuli were identical to those of Experiment 1, except that we removed the panels’ cast shadows (Fig 3). The mean of Michelson contrast was 92.04% (*SD* = 14.34) for natural condition without context and 92.62% (*SD* = 13.80) for unnatural condition.

#### Apparatus, procedure, and data coding

The apparatus, procedure, and data coding were the same as those in Experiment 1. To calculate interobserver agreement, a second observer’s measurement of the infants’ looking time was obtained for 31.25% of the total data. An interobserver agreements was *r* = .988.

### Results

#### Familiarization trials

For the familiarization phase, individual looking times were averaged across the first three and the last three trials (Table 1). We performed a 2×2 ANOVA for the looking times, with (i) trial (the first three or the last three) as a within-participant factor and (ii) the infant’s age group (5- to 6- month-olds or 7- to 8-month-olds) as between-participants factors. The ANOVA revealed a significant main effect of trial: *F*(1,30) = 29.15, *p* < 0.0001, η_p_^2^ = 0.49, which reflects a decrease in looking times over the trials. No other effects were reliable: *F*(1,30) = 0.48, *p* = 0.50, η_p_^2^ = 0.02 (main effect of age group); *F*(1,30) = 0.14, *p* = 0.71, η_p_^2^ = 0.005 (interaction of trial and age group).

#### Test trials

The mean total looking times of each age group were 22.59 s for the younger infants, and 21.02 s for the older ones (Table 2). In the natural condition, the mean total looking time was 22.47 s for 5- to 6-month-olds, and 21.31 s for 7- to 8-month-olds. In the unnatural condition, the mean total looking time was 22.71 s for 5- to 6-month-olds, and 20.74 s for 7- to 8-month-olds.

**Table 2:** Mean total looking times (in seconds) in the familiarization trials and the test trials. Standard deviations are given in parentheses.

	Familiarization trials				Test trials (max = 30s)	
	First three trials (max = 15s)		Last three trials (max = 15s)			
Age group	Mean (s)	SD	Mean (s)	SD	Mean (s)	SD
5-to-6-month olds (n = 16)	13	1.25	10.18	2.99	22.59	4.72
7-to-8-month olds (n = 16)	12.25	2.1	9.8	3.47	21.02	5.68

**Fig 4:**
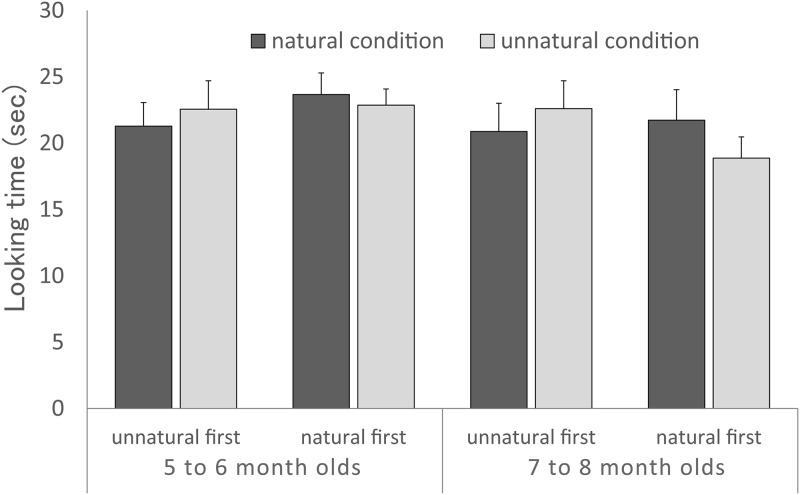
Results of Experiment 2. Mean looking times for each condition as a function of age group and presentation order. Error bars indicate standard errors.

The looking times during the test trials are depicted in Fig 4. To investigate whether infants looked longer at the unnatural condition than the natural condition, a 2×2×2 ANOVA was performed on preference scores, with (i) condition (natural or unnatural) as a within-participants factor and (ii) age group (5 to 6 months or 7 to 8 months) and (iii) order of test trial (natural condition first or unnatural condition first) as between-participants factors. No effects were reliable (*p* > 0.05): *F*(1,28) = 0.04, *p* = 0.85, η_p_^2^ = 0.001 (main effect of condition), *F*(1,28) = 0.0007, *p* = 0.98, η_p_^2^ < 0.00 (main effect of order of test trial), *F*(1,28) = 0.87, *p* = 0.36, η_p_^2^ = 0.03 (main effect of age group). These results indicate that the infants showed no preference for the natural or unnatural condition when the lightness change of the duck’s surface was not based on the cast shadow.

Additionally, we examined whether a significant difference existed in the preference for the unnatural condition across the two experiments. A three-way ANOVA was performed with age group (5- to 6- month-olds, 7- to 8- month-olds) and Experiment (Experiment 1, Experiment 2) as a between-participants factor, and condition (natural, unnatural) as a within-participants factor. The ANOVA indicated a significant three-way interaction, *F*(1, 60) = 4.90, *p* = .0.03, η_p_^2^ = 0.15. The simple-effect analysis showed that mean looking time in the unnatural condition of the older group was significantly different between Experiment 1 (25.47 ms) and Experiment 2 (20739.12 ms): *F*(1, 120) = 7.25, *p* =. 008, η_p_^2^ = 0.14. Also, mean looking time in the unnatural condition of Experiment 1 was significantly different between the younger (25470.06 ms) and the older (20.74 s) group: *F*(1, 120) = 6.53, *p* = .011, η_p_^2^ = 0.14. Additionally, the older group’s mean looking time in Experiment 1 was significantly different in the natural (25.47 s) and the unnatural (20.74 s) condition: *F*(1, 60) = 12.04, *p* = .001, η_p_^2^ = 0.14, signaling their preference for the unnatural condition. No other effects were reliable: all *p* > .05. These results confirm that 7- to 8-month-old infants preferred the unnatural condition with the cast shadow.

## Discussion

We conducted two experiments to examine whether 5- to 8-month-old infants could detect an unnatural change in surface lightness produced by cast shadows. In Experiment 1, we created two types of CG movies in which a duck passed through two cast shadows. In one movie the lightness of the duck’s surface was reduced when the duck passed through the cast shadows; in the other, we reversed the lightness of the duck’s surface inside and outside the shadows to create an unnaturalness. In this movie, the duck’s surface was lighter in the shadows and darker outside of the shadows. We tested whether infants would show a preference for the unnatural lightness changes. The results of Experiment 1 showed that only 7- to 8-month-old infants showed such a preference, suggesting that 7- to 8-month-old infants could detect unnatural lightness changes in the moving duck. In Experiment 2, we created the same movies without cast shadows to test whether the preference observed in Experiment 1 was based simply on lightness changes regardless of the shadow context. No preference was observed in Experiment 2. Taken together, Experiments 1 and 2 support the conclusion that 7- to 8-month-old infants can detect unnatural changes in surface lightness from cast shadows. Our results highlight the possibility that 7- to 8-month-olds rely on the same assumption that adults use, that the lightness of an object’s surface is reduced when the object is in shadow. The novel finding from our data is that even in infancy, the visual system can infer that cast shadows dim an object’s surface. Our result, of acquisition of shadow perception within about 7 months of birth, indicates that early experiences affect development of shadow perception. In an animal study, early visual experience affected chickens’ shading perception [17]. The study showed that chicks raised in a “light-from-below” situation from birth perceived the depth from shading based on an inverted “light-from-above” assumption. This result has not been replicated [18], so the effect of early visual experience on the development of shadow perception is still under debate. However, many studies have revealed that human infants’ perception related to shadows develops around 6 months after birth. It appears safe to conclude that the first half year after birth is an important time for development of shadow perception.

Regarding shadow perception, previous studies have shown that the human visual system has some assumptions: (i) that objects are lit from above [19–21]), and (ii) that the light source is static [22]. Previous studies have shown that the ability to use these two assumptions is acquired in infancy [23, 24]. Granrud et al. [23] found that 7-month-old infants could perceive depth from shading based on the light-from-above assumption. They compared the reaching preference for shading between the binocular and the monocular condition by using luminance-gradient shading figures that were perceived as concave or convex. In their study, 7- month-olds but not 5-month-olds could perceive depth from shading based on the light-from-above assumption. Imura et al. [24] also showed evidence that infants use assumptions (i) and (ii). They examined whether infants could use these assumptions to discriminate the difference in an object’s motion trajectory from the“ball in box illusion”[22]. They showed that 6- to 7-month-old infants could discriminate the two types of ball-in-box illusion caused by the motion of a cast shadow, but they could not discriminate them when the cast shadow of the ball was located above the ball. Their findings suggest that 6- to 7-month-old infants could perceive the object’s motion trajectory by using a cast shadow and the two assumptions. In sum, previous studies concur that the ability to use the two assumptions is acquired at 6 to 7 months of age [23, 24], implying that this age is important for the development of cast shadow perception. Our study revealed that 7- to 8-month-old (but not 5- to 6-month-old) infants were also using the assumption that the surface of an object is darker when it is in shadow; therefore, our study supports and extends those previous findings.

In our experiments, we used infants’ preference for unnaturalness to test whether they would prefer the unnaturalness of an object’s lightness in shadows. Previous studies [13–15] have used the unnaturalness preference to examine infants’ perception of illusory contours and 3D structures.

Van de Walle et al. [16] used unnatural preference to examine 5- and 8- month-old infants’ perception of synchronized motion of an object and its cast shadow. As far as we know, their study is the first to investigate infants’ cast shadow perception; however, their finding of 5-month-old infants’ sensitivity to cast shadow was not replicated in later studies [24–27]. In the current study, we revealed that 7- to 8- month-old infants showed a preference for the unnatural lightness change of a duck in cast shadows, suggesting that infants at this age, at least, can detect a violation of their assumption that cast shadow cause the surface of an object to dim.

It is known that the assumption of lightness constancy is acquired at an age of about 4 months [3–5]. In Chien et al. [5], 4-month-old infants could discriminate subjective lightness on the basis of lightness constancy. Our data indicate that 7- to 8-month-old infants were able to assume that lightness changes in an object when it passes through shadows. Taken together, Chien’s and our results suggest that although lightness constancy is acquired by 4 months of age, the ability to use it to perceive an object's lightness in shadows may not be acquired until around 7 to 8 months. We believe that stable perception of an object’s lightness is necessary for perceiving the world as constant.

Our results only imply that infants can perceive as unnatural the lightening of an object’s surface when it enters shadow. In adults, the visual system automatically compensates for the influence of shadows on the lightness of an object’s surface [2, 28]. A conspicuous example of such a visual system was shown in Adelson’s checker shadow illusion [28]. The current study did not show evidence for such compensation in infancy. Future studies will be needed to explore whether infants can perceive an object’s lightness based on such a compensation for the shadow’s influence.

## Supporting information

S1 Dataset Raw data of the looking times of valid trials for both experiments (Experiment 1 and 2). (XLSX)

S1 File Examples of the natural condition in Experiment 1. (MP4)

S2 File Examples of the unnatural condition in Experiment 1. (MP4)

S3 File Examples of the familiarization in Experiment 1. (MP4)

S4 File Examples of the natural condition without shadows in Experiment 2. (MP4)

S5 File Examples of the unnatural condition without shadows in Experiment 2. (MP4)

S6 File Examples of the familiarization without shadows in Experiment 2. (MP4)
